# Influence of renal insufficiency on anticoagulant effects and safety of warfarin in Chinese patients: analysis from a randomized controlled trial

**DOI:** 10.1007/s00210-020-02037-3

**Published:** 2021-01-06

**Authors:** Xiaoyi Ning, Yun Kuang, Guoping Yang, Jinlian Xie, Da Miao, Chengxian Guo, Zhijun Huang

**Affiliations:** 1 Center for Clinical Pharmacology, the Third Xiangya Hospital, Central South University, 138 TongZiPo Road, Changsha, 410013 Hunan China; 2 Research Center for Drug Clinical Evaluation of Central South University, Changsha, 410013 Hunan China; 3 Department of Pharmacy, the Third Xiangya Hospital, Central South University, Changsha, 410013 Hunan China; 4 Department of Nephrology, the Third Xiangya Hospital, Central South University, Changsha, 410013 Hunan China

**Keywords:** Warfarin, Anticoagulants, Chronic kidney disease, Thromboembolism

## Abstract

This study aimed to analyze the influence of renal insufficiency on the anticoagulant effects and safety of warfarin in Chinese patients. Data on the creatinine levels of participants enrolled in a randomized controlled study were screened and divided into the non-renal insufficiency group, mild renal insufficiency group, and moderate renal insufficiency group, according to the creatinine clearance rate. The primary outcome measures were stable dose and average daily dose of warfarin. Secondary outcome measures were percentage of time in the therapeutic international normalized ratio (INR) (%TTR), and the first time to reach the therapeutic INR. Adverse events included bleeding events, thromboembolic events, and mortality. All participants with renal function test results and a baseline INR of less than 1.5 were included in the primary and secondary outcome analysis. The SPSS Statistics 21.0 software was used for statistical analysis. The randomized controlled trial was registered in Clinicaltrials.gov (NCT02211326). A total of 571 patients were included in this analysis. Multiple regression analysis showed that the renal function was correlated with stable dose, average daily dose, and the first time to reach therapeutic INR after adjusting for confounding factors. However, no correlation was noted between kidney function and %TTR. No significant differences were observed across the various safety parameters among the three groups. Renal function is an important consideration in patients using warfarin.

**Supplementary Information:**

The online version contains supplementary material available at 10.1007/s00210-020-02037-3.

## Background

Warfarin is the most common and effective anticoagulant, used to prevent and treat thromboembolic disease worldwide. However, a particular challenge associated with the use of warfarin is its narrow therapeutic index with large individual variations in the daily dose requirement, often leading to either insufficient or excessive anticoagulation (Wysowski et al. 2007). Warfarin is a racemate consisting of S*-*warfarin and R*-*warfarin. S*-*warfarin, which exerts the main anticoagulant effect, is mainly metabolized by CYP2C9 into S*-6* and S*-7* hydroxyl products. R*-*warfarin is mainly metabolized by CYP1A2, CYP3A4, and CYP2C19 into R-8 warfarin. Warfarin is almost entirely metabolized by the liver. The hydroxyl products, which have weak anticoagulant effects, are mainly excreted by the kidney, and only a small amount of the prototype drug is excreted in the urine. Theoretically therefore, no dose adjustment of warfarin is needed in patients with chronic kidney disease (CKD). The warfarin prescribing information does not specify dosage recommendations guided by renal function.

As a global health problem, CKD is closely related to cardiovascular disease. The prevalence of any cardiovascular disease is twice as high in patients with CKD in the USA, based on the latest kidney data system report (Usrds 2018). The prevalence of atrial fibrillation (AF) in patients with CKD is 12–18%, including 7–8% in the general population over 65 years of age. Previous studies have shown that renal insufficiency is independently related to bleeding risk in patients with AF, who are treated with warfarin (Hirai et al. 2017; Jun et al. 2017). Renal insufficiency is also an independent risk factor in the HAS-BLED score for evaluating the bleeding risk of warfarin anticoagulation (Pisters et al. 2010). Therefore, the coexistence of high coagulation and high bleeding risk is a high risk factor in patients with CKD who also use warfarin. Previous studies have demonstrated that CKD is an independent risk factor for AF and is associated with a higher risk of stroke (Bonde et al. 2014).

The pathological state of renal insufficiency can affect the systemic exposure of renally eliminated drugs, which, in turn, affects the efficacy of those drugs and can lead to an increase in the number of adverse reactions. However, recent clinical studies have found that renal insufficiency affects not only the internal exposure of renally eliminated drugs but also *in vivo* exposure of non-renally eliminated drugs. A previous study reported a 50% increase in the plasma warfarin S/R ratio among patients with end-stage renal disease (ESRD), compared with those without ESRD after accounting for the CYP2C9 genotype (Dreisbach et al. 2003). Another clinical study showed that CKD could inhibit the metabolism of (S)-warfarin and (R, S)-warfarin (Albrecht et al. 2017).

However, as previously mentioned, no dosage recommendations in the prescribing information for warfarin have been based on renal insufficiency. Moreover, the safety and effectiveness of warfarin in patients with renal insufficiency are also controversial (Altawalbeh et al. 2018). The results of a prospective cohort study showed that in patients with AF and acute myocardial infarction, warfarin was associated with a lower 1-year risk of the composite endpoint events of mortality, myocardial infarction, and ischemic stroke in 1 year, without a higher risk of bleeding (Carrero et al. 2014). Similar findings were observed in patients with renal insufficiency, who showed event outcomes that were not associated with the degree of renal function. Another study showed that anticoagulation over the targeted international normalized ratio (INR) values is associated with a steeper decline in the estimated glomerular filtration rate and an increased frequency of CKD in patients with a mechanical prosthetic valve (Canga et al. 2018).

At present, no large randomized controlled clinical trials have been conducted to support the use of warfarin in patients with CKD. Furthermore, the existing warfarin anticoagulation guidelines for patients with CKD are derived mostly from retrospective studies. For patients with CKD, the risk–benefit ratio of warfarin needs to be carefully considered, because of the increased risk of both stroke and bleeding (Chang et al. 2019; Olesen et al. 2012; Potpara et al. 2018; Zhang et al. 2019).

Therefore, the current study, based on a prospective randomized controlled trial, analyzed the effects of renal insufficiency on anticoagulant dose and safety in Chinese patients with AF or deep venous thrombosis while using warfarin. The results should provide a reference for the reasonable use of warfarin in patients with CKD.

## Methods

### Study design

This analysis was based on a prospective randomized controlled study registered in Clinicaltrials.gov (NCT02211326) (Guo et al. 2020). Patients from 15 hospitals in China (Table S1 in Supplement 1) were recruited to participate in this multicenter, randomized, single-blind, parallel-controlled study. The study was approved by the independent ethics committee of the Third Xiangya Hospital of Central South University with an Association for the Accreditation of Human Research Protection Programs, Inc. (AAHRPP), accreditation and the independent ethics committee of each participating hospital. All participants provided written informed consent prior to the trial. Safety data were reported to and reviewed by an independent Data Safety Monitoring Board.

A flow chart of the study design is provided in Figure S1 in Supplement 2. Collection of relevant data and examination of routine biochemical indicators (liver and kidney function, whole blood count, urine analysis, and routine stool analysis) were performed by designated researchers at each participating hospital. The INR values were detected on a Roche Coaguchek XS system. Data were analyzed and independently confirmed by three statisticians. All researchers vouched for study protocol adherence. Genotyping for the CYP2C9*2, CYP2C9*3, and *VKORC1-1639G>A* alleles was performed using the amplification refractory mutation system (ARMS), which provided genotype results in approximately 4 h (Zhu et al. 2010). The genotyping of all samples was validated by Sanger sequencing.

### Study participants

We recruited patients who were 18 years or older, with AF or deep vein thrombosis (DVT). Patients who had received previous treatment with warfarin, or who had a high bleeding risk were excluded. Furthermore, patients with a hemorrhagic tendency, who were planning to undergo an invasive examination, or surgery during the trial, and those for whom clinical judgment predicted outcomes of bleeding were also excluded. The detailed inclusion and exclusion criteria are presented in Table S2 in Supplement 1.

### Procedures

The dosing regimen was randomly divided into genotype-guided (the first 3 days according to the IWPC [International Warfarin Pharmacogenetics Consortium] formula, the fourth to seventh days, according to the Lenzini formula (Lenzini et al. 2010), followed by adjustment by the clinician based on the INR), and clinical experience (the first 3 days 2.25 mg/day, followed by adjustment by the clinician based on the INR). The dosage adjustment regulations are presented in Table S3 and Table S4 in Supplement 1.

The study period was 12 weeks, with a baseline visit and eight follow-up visits as follows: − 3 to − 1 (baseline measurements prior to dosing); 1; 4/5; 8 ± 1; 15 ± 1; 22 ± 1; 28 ± 2; 57 ± 3; and 87 ± 3 days. Some participants had additional clinic visits and INR measurements based on clinical needs. The dosing algorithms and a detailed flow chart of follow-up visits are provided in Figure S1, included in Supplement 1.

The participants with creatinine data were screened and divided into the non-renal insufficiency group, mild renal insufficiency group, and moderate renal insufficiency group, according to the creatinine clearance rate. Renal function was graded as follows: non-renal insufficiency group, CrCL creatinine clearance ≥ 90 mL/min; mild renal insufficiency group, 60 ≤ CrCL < 90 mL/min; moderate renal insufficiency, 30 ≤ CrCL <60 mL/min. The formula for CrCL was as follows: CrCL = [(140 − age) × weight (kg)]/[0.818 × creatinine (μmol/L)] for men, and CrCL = [(140 − age) × weight (kg)]/[0.818 × creatinine (μmol/L)] × 0.85 for women.

### Outcome measures

The primary outcome measures were stable dose of warfarin, defined as the dose to achieve the INR within ± 0.1 of the therapeutic range at day 8 after dosing for 2 consecutive weeks, and the average daily dose. Secondary outcome measures were percentage of time in the therapeutic INR (%TTR) and the first time to reach therapeutic INR. Adverse events included bleeding events (mild, moderate, or severe) (Rosendaal et al. 1993), thromboembolic events, and mortality.

### Statistical analysis

All participants with renal function test results and a baseline INR of less than 1.5 were included in the primary and secondary outcome analyses. Furthermore, additional subgroup analyses for different *CYP2C9* and *VKORC1* genotypes were performed. Three groups were defined based on the United States Food and Drug Administration (FDA) genotype-based dosing recommendations as follows. Highly Sensitive responder: CYP2C9*1/*3 and *VKORC1 AA*, CYP2C9*3/*3 and *VKORC1 AA* or *GG* or *GA*. Sensitive responder: CYP2C9*1/*1 and *VKORC1 AA*, CYP2C9*1/*3 and *VKORC1 GG* or *GA*. Normal responder: CYP2C9*1/*1 and *VKORC1 GG* or *GA*.

All of the statistical indicators were selected for complete data analysis. The SPSS Statistics 21.0 software was used for statistical analysis, and the significance level was set at *P* ≤ 0.05. Unless otherwise specified, measurement data were expressed as mean ± SD. The Kruskal–Wallis test was applied for measurement data. Count data were expressed by direct notation or as a percentage, and the difference between groups was compared using the chi-square test, Fisher’s exact test, or continuity correction chi-square test. The chi-square test was also used to evaluate test whether the frequencies of genes and alleles were consistent with Hardy–Weinberg equilibrium.

Renal function was considered an ordinal categorical variate, as follows: non-renal insufficiency group = 1; mild renal insufficiency group = 2; moderate renal insufficiency group = 3. Multivariable analysis was used to estimate the effect of renal function on outcome variables after controlling for the confounding effects of other variables. The confounding variables included age, sex, weight, baseline INR, indications, and mode of administration. Multiple linear regression was used to analyze the continuous variables, and logistic regression was used to analyze the binary variables.

## Results

### Participants

We selected 571 patients with renal function results from the 660 patients in the previous randomized controlled study, and then classified them into the non-renal insufficiency group (*n* = 77), mild renal insufficiency group (*n* = 235), and moderate renal insufficiency group (*n* = 259) according to renal function. The demographics and baseline clinical characteristics of the study participants are shown in Table 1. With the exception of age (*P* < 0.001), height (*P* < 0.001), weight (*P* < 0.001), baseline INR (*P* = 0.029), and indications (*P* < 0.001), no statistical differences were observed in other indicators. Patients with weaker renal function tended to be older, shorter, and leaner; have a higher baseline INR; and more frequently had AF than DVT. In addition, the genotypic distributions of *CYP2C9* and *VKORC1* conformed with Hardy–Weinberg equilibrium(*P* > 0.05).

**Table 1:** Demographic and baseline clinical characteristics of the study participants

Index	Non-renal insufficiency group (*n* = 77) (Mean ± SD)	Mild renal insufficiency group (*n* = 235) (Mean ± SD)	Moderate renal insufficiency group (*n* = 259) (Mean ± SD)	*P*
Age (years)^a^	55.82 ± 9.52	65.17 ± 9.07	72.24 ± 7.21	< 0.001*
Height (cm)^a^	1.64 ± 0.07	1.62 ± 0.08	1.60 ± 0.08	< 0.001*
Weight (kg)^a^	71.61 ± 11.66	63.82 ± 11.06	58.12 ± 11.07	< 0.001*
Baseline INR^a^	1.02 ± 0.11	1.04 ± 0.10	1.05 ± 0.10	0.029
Sex, *n* (%)^b^				
Male	46 (59.7)	122 (51.9)	123 (47.5)	0.157
Female	31 (40.3)	113 (48.1)	136 (52.5)	
Indications, *n* (%)^b^				
Atrial fibrillation	56 (72.7)	205 (87.2)	234 (90.3)	< 0.001*
Deep vein thrombosis	21 (27.3)	30 (12.8)	25 (9.7)	
Mode of administration, *n* (%)^b^				
Genotype-guided dosing group	45 (58.4)	104 (44.3)	133 (51.4)	0.067
Clinical experience-guided dosing group	32 (41.6)	131 (55.7)	126 (48.6)	
Nationality, *n* (%)^d^				
Han	76 (98.7)	234 (99.6)	259 (100.0)	0.231
Minority	1 (1.3)	1 (0.4)	0 (0.0)	
*CYP2C9*, *n* (%)^d^				
*1/*1	72 (93.5)	216(91.9)	240 (92.7)	0.975
*1/*3	5 (6.5)	18(7.7)	18 (6.9)	
*3/*3	0 (0.0)	1(0.4)	1 (0.4)	
*VKORC1*, *n* (%)^d^				
*AA*	59 (76.6)	185 (78.7)	216 (83.4)	0.550
*AG*	17 (22.1)	47 (20.0)	39 (15.1)	
*GG*	1 (1.3)	3 (1.3)	4 (1.5)	
Combined use of drugs, *n* (%)				
Fluvastatin^c^	0 (0.0)	3 (1.3)	4 (1.5)	0.555
Amiodarone^c^	1 (1.3)	4 (1.7)	2 (0.8)	0.643
Enzyme inducer	0 (0.0)	0 (0.0)	0 (0.0)	1.000
Co-morbidity, *n* (%)				
Diabetes^b^	12 (15.6)	36 (15.3)	35 (13.5)	0.818
Hypertension^b^	37 (48.1)	125 (53.2)	135 (52.1)	0.735
Apoplexy ^c^	1 (1.3)	11 (4.7)	11 (4.2)	0.412

*P < 0.05, with statistical significance

^a^Kruskal–Wallis test

^b^Chi-square test

^c^Continuity correction chi-square test

^d^Fisher’s exact test

### Primary outcome measure

According to the analysis of renal function in 396 participants, the stable dose was 3.12 ± 1.04 mg in the non-renal insufficiency group, 2.58 ± 0.91 mg in the mild renal insufficiency group, and 2.10 ± 0.80 mg in the moderate renal insufficiency group (Table 2). Multiple regression analysis showed that renal function was correlated with the stable dose after adjusting for confounding factors (Table 3). With increasing severity of renal insufficiency, the stable dose of warfarin required was reduced.

**Table 2:** Descriptive statistics of primary and secondary outcomes among participants with different renal functions

Measures	Non-renal insufficiency group		Mild renal insufficiency group		Moderate renal insufficiency group	
	*N*	Mean ± SD	*N*	Mean ± SD	*N*	Mean ± SD
Primary outcomes						
Stable dose (mg)	49	3.12 ± 1.04	173	2.58 ± 0.91	174	2.10 ± 0.80
Average daily dose (mg)^a^	77	3.21 ± 1.09	235	2.68 ± 0.84	259	2.24 ± 0.67
Secondary outcomes						
Percentage of time in therapeutic INR range (%TTR)^b^						
1–4/5 days	72	3.60 ± 11.66	228	7.98 ± 17.38	247	12.96 ± 21.32
1–8 days	72	19.83 ± 22.50	222	29.28 ± 23.71	239	35.63 ± 21.88
1–15 days	68	36.67 ± 25.78	210	40.81 ± 23.10	224	40.64 ± 21.20
1–22 days	66	42.90 ± 28.21	206	46.09 ± 23.23	209	44.40 ± 22.53
1–28 days	65	46.15 ± 28.06	202	50.63 ± 23.62	206	48.01 ± 22.53
1–57 days	63	47.85 ± 29.60	194	57.40 ± 25.18	203	57.45 ± 24.00
1–87 days	59	49.26 ± 27.93	186	59.56 ± 25.13	190	61.64 ± 24.91
The first time to reach therapeutic INR	68	11.87 ± 14.39	215	7.27 ± 6.89	239	5.63 ± 3.51

^a^Average daily dose = total dose during the follow-up period/total follow-up days

^b^%TTR was calculated by linear interpolation and cubic spline interpolation

*INR*, international normalized ratio; *%TTR*, percentage of time in therapeutic INR

**Table 3:** Multiple linear regression analysis of stable dose

Covariates	Regression coefficient	Std. error	*P*
Intercept	4.451	0.576	< 0.001*
Renal functions	− 0.223	0.080	0.006*
Age	− 0.183	0.083	0.028*
Weight	− 0.016	0.005	0.003*
Baseline INR	0.018	0.004	< 0.001*
Sex	− 0.893	0.444	0.045*
Mode of administration	0.033	0.095	0.732
Indications	− 0.591	0.130	< 0.001*

*P < 0.05, with statistical significance

*INR*, international normalized ratio

In addition, 571 participants received an average daily dose. The average daily dose was 3.21 ± 1.09 mg in the non-renal insufficiency group, 2.68 ± 0.84 mg in the mild renal insufficiency group, and 2.24 ± 0.67 mg in the moderate renal insufficiency group (Table 2). Multiple regression analysis showed that renal function was correlated with the average daily dose after adjusting for confounding factors (Table 4). The average daily dose in the non-renal insufficiency group was higher than that in both the mild renal insufficiency group and moderate renal insufficiency group.

**Table 4:** Multiple linear regression analysis of average daily dose

Covariates	Regression coefficient	Std. error	*P*
Intercept	4.861	0.441	< 0.001*
Renal functions	− 0.220	0.061	< 0.001*
Age	− 0.036	0.064	0.577
Weight	− 0.016	0.004	< 0.001*
Baseline INR	0.013	0.003	< 0.001*
Sex	− 1.152	0.327	< 0.001*
Mode of administration	0.057	0.073	0.433
Indications	− 0.414	0.098	< 0.001*

*P < 0.05, with statistical significance

*INR*, international normalized ratio

### Secondary outcome measures

The multivariable analysis showed no correlation between kidney function and %TTR, whereas renal function was correlated with the first time to reach therapeutic INR (Table 5). The first time to reach therapeutic INR values in the non-renal insufficiency group, the mild renal insufficiency group, and the moderate renal insufficiency group were 20.00 ± 15.31 days, 10.90 ± 12.61 days, and 6.05 ± 4.75 days respectively (Table 2). The first time to reach therapeutic INR among participants with renal insufficiency was earlier than that among participants with non-renal insufficiency. Furthermore, the renal function showed a gradual decline and the first time to reach therapeutic INR was significantly reduced, with an average difference of more than 4 days among the three groups.

**Table 5:** Multiple linear regression analysis of the first time to reach therapeutic INR

Covariates	Regression coefficient	Std. error	*P*
Intercept	23.965	4.256	< 0.001*
Renal functions	− 1.373	0.598	0.022*
Age	− 2.095	0.624	0.001*
Weight	− 0.125	0.039	0.001*
Baseline INR	0.037	0.033	0.266
Sex	− 5.148	3.198	0.108
Mode of administration	− 0.180	0.707	0.799
Indications	− 1.188	0.954	0.213

*P < 0.05, with statistical significance

*INR*, international normalized ratio

### Analysis of adverse events

Adverse events were recorded throughout the study. A total of 571 participants were evaluated for safety outcome measures as follows: 77 participants from the non-renal insufficiency group, 235 participants from the mild renal insufficiency group, and 259 participants from the moderate renal insufficiency group. No significant differences were noted in overall adverse events among the three groups (Table 6). Overall, 33 bleeding events (four in the non-renal insufficiency group, 13 in the mild renal insufficiency group, and 17 in the moderate renal insufficiency group); 21 mild bleeding events (three in the non-renal insufficiency group, nine in the mild renal insufficiency group, and nine in the moderate renal insufficiency group); seven moderate bleeding events (one in the non-renal insufficiency group, one in the mild renal insufficiency group, and five in the moderate renal insufficiency group); and five severe bleeding events (three in the mild renal insufficiency group and two in the moderate renal insufficiency group) were reported. A single mortality was reported in each of mild renal insufficiency and moderate renal insufficiency groups. Just one thromboembolic event was recorded in the moderate renal insufficiency group. No significant differences were noted across the various safety parameters among the three groups (Table 6).

**Table 6:** Safety analysis

Analysis	Non-renal insufficiency group	Mild renal insufficiency group	Moderate renal insufficiency group	*P*
	*N* (%)	*N* (%)	*N* (%)	
Adverse events related to warfarin	4 (5.2)	13 (5.5)	17 (6.6)	0.850
Bleeding events	4 (5.2)	13 (5.5)	16 (6.2)	0.928
Mild	3 (3.9)	9 (3.8)	9 (3.5)	0.972
Moderate	1 (1.3)	1 (0.4)	5 (1.9)	0.315
Severe	0	3 (1.3)	2 (0.8)	0.563
Deaths (included in severe bleeding events)	0	1 (0.4)	1 (0.4)	0.853
Thromboembolism events	0	0	1 (0.4)	0.547

### Subgroup analysis based on genotyping

Descriptive statistics of primary outcomes in the subgroup analysis are presented in Table 7. Renal function was correlated with the stable dose and average daily dose among sensitive responders (Tables 8 and 9). No correlations were noted among highly sensitive responders and normal responders.

**Table 7:** Descriptive statistics of primary outcomes in the subgroup analysis based on genotype^a^

Analysis	Non-renal insufficiency group		Mild renal insufficiency group		Moderate renal insufficiency group	
	*N*	Mean ± SD	*N*	Mean ± SD	*N*	Mean ± SD
Stable dose (mg)						
Highly sensitive responder	1	2.63	12	1.75 ± 0.40	9	1.44 ± 1.00
Sensitive responder	39	3.02 ± 1.00	130	2.42 ± 0.72	141	2.02 ± 0.64
Normal responder	9	3.61 ± 1.16	31	3.58 ± 1.02	24	2.86 ± 1.07
Average daily dose (mg)						
Highly sensitive responder	3	2.51 ± 0.26	16	1.92 ± 0.35	15	1.71 ± 0.66
Sensitive responder	58	3.03 ± 1.03	172	2.51 ± 0.63	205	2.16 ± 0.54
Normal responder	16	3.98 ± 1.02	47	3.57 ± 0.97	39	2.88 ± 0.85

^a^Highly sensitive responder: CYP2C9*1/*3 and *VKORC1 AA*; CYP2C9*3/*3 and *VKORC1 AA* or *GG* or *GA*; sensitive responder: CYP2C9*1/*1 and *VKORC1 AA*, CYP2C9*1/*3 and *VKORC1 GG* or *GA*; normal responder: CYP2C9*1/*1 and *VKORC1 GG* or *GA*.

**Table 8:** Multiple linear regression analysis of stable dose in sensitive responder

Covariates	Regression coefficient	Std. error	*P*
Intercept	4.211	0.502	< 0.001*
Renal functions	− 0.171	0.075	0.023*
Age	− 0.017	0.005	< 0.001*
Weight	0.018	0.004	< 0.001*
Baseline INR	− 0.756	0.382	0.049*
Sex	− 0.010	0.086	0.910
Mode of administration	− 0.253	0.075	0.001*
Indications	− 0.589	0.116	< 0.001*

*P < 0.05, with statistical significance

*INR*, international normalized ratio

**Table 9:** Multiple linear regression analysis of average daily dose in sensitive responder

Covariates	Regression coefficient	Std. error	*P*
Intercept	4.589	0.388	< 0.001*
Renal functions	− 0.127	0.057	0.027*
Age	− 0.019	0.004	< 0.001*
Weight	0.011	0.003	< 0.001*
Baseline INR	− 0.853	0.290	0.003*
Sex	0.038	0.066	0.566
Mode of administration	− 0.097	0.058	0.096
Indications	− 0.463	0.089	< 0.001*

*P < 0.05, with statistical significance

*INR*, international normalized ratio

The original data that support the findings of this study was provided in Supplement 2.

## Discussion

The results showed that patients with weaker renal function tended to be older. Epidemiological investigation has shown that the incidence of renal insufficiency patients in China is about 10% (Zhang et al. 2008). With increasing age, renal function tends to decline and the incidence of renal insufficiency increases. So, we have adjusted for age in the multivariate analysis, and the results showed that renal function was correlated with the stable dose and average daily dose after adjusting for confounding factors including age (Table 3 and Table 4). Based on above, we consider that after eliminating the interference of age, the influence of renal function on the warfarin effect is still significant. In comparison to those with non-renal insufficiency, patients with renal insufficiency tended to be shorter in height and have higher baseline INR values; however, these differences were small and could be considered to have no clinical significance. Moreover, the proportion of patients with AF in the renal insufficiency group was higher than that in the non-renal insufficiency group. This may be because renal insufficiency is an independent risk factor for AF (Pisters et al. 2010). The genotype frequency of the patients included in the current study conformed with the Hardy–Weinberg principle, and was representative of the general population of China.

According to the analysis of renal function in 396 participants who received a stable dose, as renal insufficiency became more severe, the stable dose of warfarin required was lower. These results are consistent with those of a previous study (Limdi et al. 2010a), which also showed that, with weakening renal function, the stable dose of warfarin can be reduced, with a similar trend in the average dose. Similar statistical results were observed in the subgroup analysis among sensitive patients. Although no correlation was observed, the multiple linear regression models established among highly sensitive responders and normal responders may not be reliable, owing to the small sample size. In addition, for patients with similar renal function, as their sensitivity to warfarin is increased, the stable dose and average daily dose should be decreased, which is consistent with the findings of previous genetics studies (Kamali 2006; Limdi et al. 2010b; Zhong et al. 2012). Based on the current results, we tried to establish a dose recommendation that takes into account both genotype and renal function, which we compared with the FDA genotype-based dosing recommendations (Table S5 in Supplement 1).

Patients in the renal insufficiency group reached the treatment INR earlier than those in the non-renal insufficiency group. This shows that administration of warfarin without considering renal function could allow patients with renal insufficiency to reach the treatment INR earlier, but would likely cause large fluctuations at a later stage (the levels of INR compliance in patients with different stages of renal function are showed in Figure S2 in Supplement 1).

In the current study, the initial dose for patients in the conventional administration group was 2.25 mg. The average initial doses or predicted doses for patients in the genotype-guided administration group with different renal function states were 2.68 ± 0.71 mg in the non-renal insufficiency group, 2.77 ± 0.71 mg in the mild renal insufficiency group, and 2.59 ± 0.73 mg in the moderate renal insufficiency group (Table S6 in Supplement 1). It is evident that although only the genotype was considered (without consideration of renal function) for dose prediction, the predicted dose for the patients with moderate renal insufficiency was too high (predicted error < − 20%, Table S6 in Supplement 1). Previous studies have shown that during warfarin anticoagulation therapy, controlling the %TTR to above the range of 58–65% can significantly reduce the risk of stroke and bleeding events (Connolly et al. 2008; Piccini et al. 2014; Wallentin et al. 2010, 2013).

The current study showed that with the exception of the mean %TTR in the non-renal insufficiency group, which was lower than 58% (49.5%), the %TTR of the other two groups were between 58 and 65%. This suggests that overall anticoagulation was favorable. The results of epidemiological studies have shown that the global anticoagulant compliance rate for warfarin is about 50.3%, and the compliance rate in China is only 36% (Oldgren et al. 2014). The current results are higher than those reported in the literature. This phenomenon could be attributed to the fact that we performed strict and frequent treatment monitoring and follow-up of patients in the previous randomized controlled trial. On the other hand, the current findings may be related to the genotype-guided administration of some of the patients in the randomized controlled trial, which may have increased the %TTR.

A total of 33 bleeding events were reported. One mortality was reported in each of the mild renal insufficiency and moderate renal insufficiency groups. Both cases developed cerebral hemorrhage and died of increased intracranial pressure. A previous study showed a 2.5-fold higher risk of hemorrhage among warfarin users with severe kidney impairment after accounting for genetic and clinical factors (Limdi et al. 2009). However, bleeding events in the three groups were not statistically significant in the current study. This may have been due to efficient treatment monitoring and follow-up in the previous randomized controlled trial. In addition, the current study only followed up for 3 months; thus, a shorter follow-up period would result in fewer observed bleeding events.

The results of the current study showed that the stable dose and average daily dose of warfarin can be gradually decreased with declining renal function. Based on the results, it is necessary to consider the renal function of patients when using warfarin.

## Supplementary Information

ESM 1 (DOCX 151 kb)

ESM 2 (XLSX 135 kb)

## Data Availability

The data that support the findings of this study will be available from the corresponding author upon request.
