# Supplementary material for: Influence of renal insufficiency on anticoagulant effects and safety of warfarin in Chinese patients: analysis from a randomized controlled trial
Source: Naunyn Schmiedebergs Arch Pharmacol. 2021 Jan 6;394(6):1275–83. doi: 10.1007/s00210-020-02037-3 (PMC8208902; doi:10.1007/s00210-020-02037-3)
Supplement: Supplementary file 1 — (DOCX 151 kb) [file 210_2020_2037_MOESM1_ESM.docx]

**SUPPLEMENTAL 1**

**The Influences of Renal Insufficiency on Anticoagulation Effect and Safety of Warfarin in Chinese Patients**

Xiaoyi Ning^1*^, Bachelor., Yun Kuang^1*^, M. Med., Guoping Yang^1,2,3^, Ph.D., Jinlian Xie^1^, Bachelor., Da Miao^1^, M. Med., Chengxian Guo^1,2,3#^, Ph.D., Zhijun Huang^1,4#^, M.D.

^1^ Center for Clinical Pharmacology, the Third Xiangya Hospital, Central South University, Changsha, Hunan, 410013, China

^2^ Research Center for Drug Clinical Evaluation of Central South University, Changsha, Hunan, 410013, China

^3^ Department of Pharmacy, the Third Xiangya Hospital, Central South University, Changsha, Hunan, 410013, China

^4^ Department of Nephrology, the Third Xiangya Hospital, Central South University, Changsha, Hunan, 410013, China

**^*^Xiaoyi Ning and Yun Kuang contributed equally to this work, and should be considered joint first author**

**^#^ Joint corresponding author:**

Chengxian Guo, Ph.D.

Center for Clinical Pharmacology, the Third Xiangya Hospital, Central South University, 138 TongZiPo Road, Changsha, Hunan, 410013, China;

E-mail: gchxyy@163.com

Zhijun Huang, M.D.

Center for Clinical Pharmacology, the Third Xiangya Hospital, Central South University, 138 TongZiPo Road, Changsha, Hunan, 410013, China;

E-mail: huangzj@csu.edu.cn

**Methodology**

**Table S1 15 Research Hospitals**

| Hospital number | Hospital name |
| --- | --- |
| 1 | The Third Xiangya Hospital of Central South University |
| 2 | Xiangya Hospital of Central South University |
| 3 | Hunan Provincial People's Hospital |
| 4 | Changsha Central Hospital |
| 5 | The Third Hospital of Changsha |
| 6 | The Fourth Hospital of Changsha |
| 7 | Xiangtan Central Hospital |
| 8 | The First People's Hospital of Xiangtan City |
| 9 | Shaoyang Central Hospital |
| 10 | The First People's Hospital of Shaoyang |
| 11 | The First Affiliated Hospital of the University of South China |
| 12 | The Second Affiliated Hospital of the University of South China |
| 13 | The First People's Hospital of Chenzhou |
| 14 | Yiyang Central Hospital |
| 15 | Loudi Central Hospital |

**Table S2 Inclusion and exclusion criteria**

| Inclusion criteria | Exclusion criteria |
| --- | --- |
| 1) Chinese male or female aged ≥18 years;  2) Requiring at least 12-week warfarin therapy as judged by clinicians;  3) Subjects with DVT or AF and having a post-therapy target INR of 1.5-2.5 for aged ≥60 years, having a post-therapy target INR of 2.0-3.0 for aged <60 years;  4) Capable of providing written informed consent;  5) Capable of maintaining excellent communications with investigators and completing trial in accordance with trial stipulations. | 1) Subjects previously taking warfarin;  2) Known genotypes of *CYP2C9* or/and *VKORC1*;  3) Subjects receiving or expecting to receive other therapies or other anticoagulants;  4) Subjects with contraindications for warfarin;  5) Subjects with severe cognitive dysfunctions;  6) Baseline INR ≥1.5;  7) Subjects with drug or alcohol dependence within the last 12 months;  8) Subjects receiving blood transfusion or bone marrow transplantation within the last 2 weeks;  9) Planning to receive invasive examinations (except for standard endoscopy) with a hemorrhagic tendency or undergoing surgery during trial.  10) Prior to randomization, subjects receiving any trial drug or device within the last 3 months or planning to receive such an investigational therapy during trial;  11) Subjects with the following diagnoses or conditions: active malignant carcinomas (diagnosed within the last 5 years), but excluding adequately-treated non-melanoma skin cancer or other non-invasive or *in situ* cancer (e.g. cervical cancer *in situ*); anti-neoplastic therapy within the last 5 years (medication, radiotherapy or/and surgery); overt active disease or infection; life expectancy <6 months;  12)Other clinical reasons for unsuitable recruitment as judged by clinicians. |

**Table S3 Dose adjusting recommendations based upon INR (applicable for subjects aged ≥60)**

| INR Range | Measures |
| --- | --- |
| <1.0 | Boost previous dose by 40%-50% |
| 1.0~1.4 | Boost previous dose by 20%-35% |
| 1.5~2.5 | Normal dosing |
| 2.6~3.0 | Normal dosing, strengthen monitoring and adjusting if necessary (>2.6-3.0 on at least three consecutive occasions, lowering previous dose by 20%-35%) |
| 3.0~4.5 | Lower previous dose by 20%-35% |
| ≥4.5 | 1. Inquire about hemorrhagic tendency if necessary 2. Pause dosing for 2 days, inject intramuscularly vitamin K1 (1.0-2.5 mg), re-examine INR at 6-12h and restart 2.25 mg warfarin therapy when INR<3. |
| Severe hemorrhage (regardless of INR level) | Withdraw warfarin, inject intramuscularly vitamin K1 (5 mg), infuse fresh frozen plasma, prothrombin concentrate or recombinant coagulation factor VIIa and monitor INR real-time. Upon disease stabilization, re-evaluate the necessity of applying warfarin therapy. |

**Table S4 Dose adjusting recommendations based upon INR (applicable for subjects aged <60)**

| INR range | Measures |
| --- | --- |
| <1.0 | Boost previous dose by 40%-50% |
| 1.0~1.9 | Boost previous dose by 20%-35% |
| 2.0~3.0 | Normal dosing |
| 3.0~4.5 | Lower previous dose by 20%-35% |
| ≥4.5 | 1. Inquire about hemorrhagic tendency if necessary 2. Pause dosing for 2 days, inject intramuscularly vitamin K1 (1.0-2.5 mg), re-examine INR at 6-12h and restart 2.25 mg warfarin therapy when INR<3 |
| Severe hemorrhage (regardless of INR level) | Withdraw warfarin, inject intramuscularly vitamin K1 (5mg), infuse fresh frozen plasma, prothrombin concentrate or recombinant coagulation factor VIIa and monitor INR real-time. Upon disease stabilization, re-evaluate the necessity of applying warfarin therapy. |

Note: Vitamin K1 may be administered i.v., s.c. or p.o.. Intravenous injection of vitamin K1 is predisposed to cause hypersensitive reactions while the onset of action is relatively slow for oral vitamin K1. When emergency reversion of anti-coagulation is needed, vitamin K1 is slowly injected intravenously. When continuing warfarin therapy after using a large dose of vitamin K1, heparin is dosed until the effect of vitamin K1 disappears and patients restore the responses to warfarin therapy.

**Figure S1 Dosing scheme**

Consenting patient

Genotype-guided group

Control group

Randomization

PG-1 dose

Routine dose 2.25mg

PG-1 dose

PG-2 dose

Routine dose 2.25mg

Adjust dose according to INR

Adjust dose according to INR

剂量

Statistical analysis

Genotype unavailable at day 1

D1

D2~3

D4/5

D8~D90

D-3~-1

**Discussion**

**Table S5 Comparison of dose recommendation between FDA genotype-based dosing recommendations and the study’ s results under different renal function (unit: mg)**

| **Index** | ***CYP2C9*** | ***VKORC1-1639G>A*** | | |
| --- | --- | --- | --- | --- |
|  |  | ***GG*** | ***GA*** | ***AA*** |
| Non-renal insufficiency | ****1*1*** | 3.61±1.16 | 3.61±1.16 | 3.02±1.00 |
|  | ****1*3*** | 3.02±1.00 | 3.02±1.00 | 2.63 |
|  | ****3*3*** | 2.63 | 2.63 | 2.63 |
| Mild renal insufficiency | ****1*1*** | 3.58±1.02 | 3.58±1.02 | 2.42±0.72 |
|  | ****1*3*** | 2.42±0.72 | 2.42±0.72 | 1.75±0.40 |
|  | ****3*3*** | 1.75±0.40 | 1.75±0.40 | 1.75±0.40 |
| Moderate renal insufficiency | ****1*1*** | 2.86±1.07 | 2.86±1.07 | 2.02±0.64 |
|  | ****1*3*** | 2.02±0.64 | 2.02±0.64 | 1.44±1.00 |
|  | ****3*3*** | 1.44±1.00 | 1.44±1.00 | 1.44±1.00 |
| FDA genotype-based dosing recommendations | ****1*1*** | 5-7 | 5-7 | 3-4 |
|  | ****1*3*** | 3-4 | 3-4 | 0.5-2 |
|  | ****3*3*** | 0.5-2 | 0.5-2 | 0.5-2 |

**Figure S2 Trend of INR compliance over time among participants with different renal functions**

**
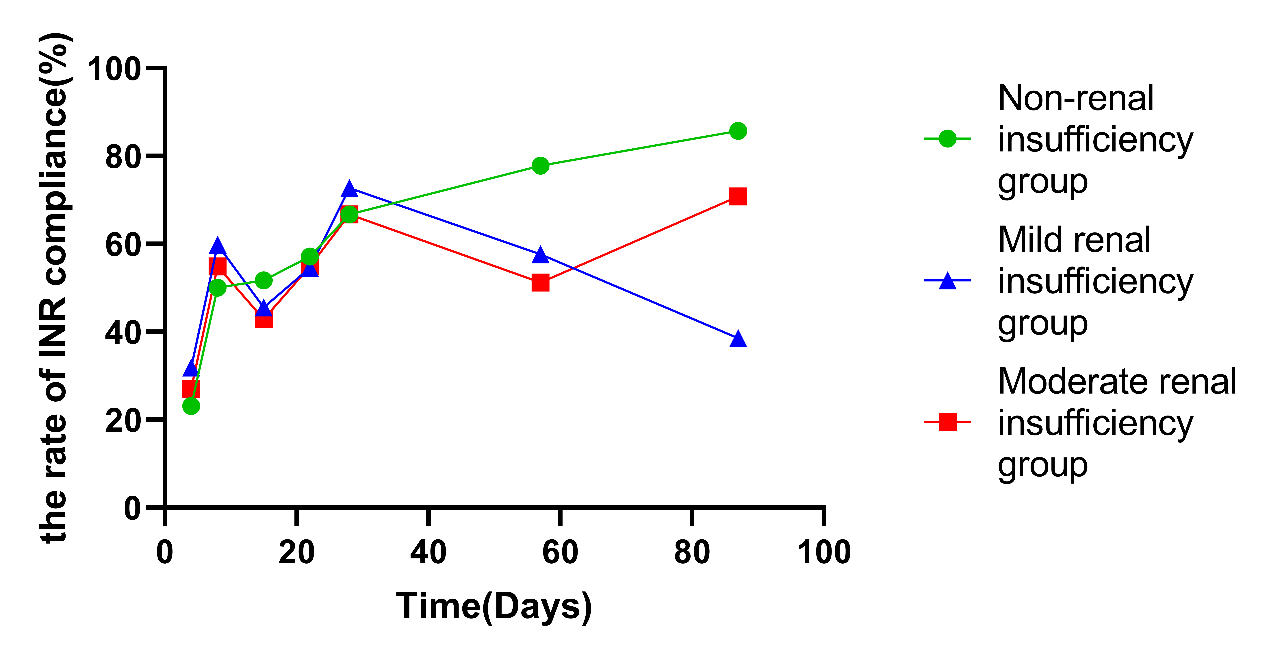
**

**Table S6 Comparison of stable doses and predicted dose by IWPC formula under different renal function**

| Measurement (mg) | Non-renal insufficiency | | Mild renal insufficiency | | Moderate renal insufficiency | |
| --- | --- | --- | --- | --- | --- | --- |
|  | N | Mean ± SD | N | Mean ± SD | N | Mean ± SD |
| Stable dose | 49 | 3.12±1.04 | 173 | 2.58±0.91 | 174 | 2.10±0.80 |
| Predicted dose | 37 | 2.68±0.73 | 115 | 2.77±0.67 | 130 | 2.59±0.73 |
| Prediction error* |  | 14.10% |  | -7.36% |  | -23.33% |

* Prediction error：(predicted dose-stable dose) / stable dose * 100%, the prediction accuracy of the IWPC formula: predicted error is within ±20%
